# Supplementary material for: Cellular-resolution mapping uncovers spatial adaptive filtering at the rat cerebellum input stage
Source: Commun Biol. 2020 Oct 30;3:635. doi: 10.1038/s42003-020-01360-y (PMC7599228; doi:10.1038/s42003-020-01360-y)
Supplement: Supplementary file 3 — Description of Additional Supplementary Files [file 42003_2020_1360_MOESM3_ESM.pdf]

Description of additional supplementary files

File name: Supplementary Data 1

Description: Source data for all main figures.

File name: Supplementary Movie 1.

Description: Activation of a response unit (inhibition ON). The unit is the same as in Fig. 6 and is activated by 50 mossy fibers. Small dots are granule cells, large dots are Golgi cells that participate to inhibit the granule cells of the unit. Spike generation is evidenced by cell lightening.

File name: Supplementary Movie 2.

Description: Activation of a response unit (inhibition OFF). The unit is the same as in Fig. 6 and is activated by 50 mossy fibers. Small dots are granule cells, large dots are Golgi cells that participate to inhibit the granule cells of the unit. Spike generation is evidenced by cell lightening. Note wider and stronger activation of the unit compared to inhibition ON (Supplementary Movie 1).

File name: Supplementary Movie 3.

Description: Activation of a response unit after plasticity (inhibition ON). The unit is the same as in Fig. 6 and is activated by 50 mossy fibers. Plasticity was induced by applying the Bear-Lisman rule. Small dots are granule cells, large dots are Golgi cells that participate to inhibit the granule cells of the unit. Spike generation is evidenced by cell lightening. Note wider and stronger activation compared to control (Supplementary Movie 1).

File name: Supplementary Movie 4.

Description: Activation of a response unit after plasticity (inhibition OFF). The unit is the same as in Fig. 6 and is activated by 50 mossy fibers. Plasticity was induced by applying the Bear-Lisman rule. Small dots are granule cells, large dots are Golgi cells that participate to inhibit the granule cells of the unit. Spike generation is evidenced by cell lightening. Note wider and stronger activation compared to control (Supplementary Movie 2).
